# 

**Published:** 1882-11

**Authors:** 


CONTENTS.

| | Page. |
|---|---|
| Face Ache | 491 |
| Too Fat | 492 |
| Tight Lacing | 493 |
| Typhoid Fever and Salicylic Acid | 494 |
| Snake Bites | 496 |
| "Though Lost to Sight, to Memory Dear" | 497 |
| To Stop Bleeding | 497 |
| How Consumption may be Prevented | 498 |
| Mississippi Floods | 500 |
| The Use of Tobacco by Boys | 501 |
| House and Garden | 502 |
| Great Things | 503 |
| Africa's Population | 504 |
| Labor and Capital | 505 |
| Germs of Disease | 506 |
| Arctic Mosquitoes | 507 |
| The Timber Supply | 509 |
| About Bees | 511 |
| Mining in Hot Places | 512 |
| How to Mesmerize | 513 |
| Norwegian Grave Yards | 515 |
| Largest Cotton Planter in the World | 516 |
| A Strange Country | 517 |
| Toothpicks | 518 |
| Lincoln as a Peacemaker | 519 |
| Tremors of the Earth | 520 |
| The Stinging Tree | 520 |
| Two Funny Doctors | 520 |

